# *IL2RG* hypomorphic mutation: identification of a novel pathogenic mutation in exon 8 and a review of the literature

**DOI:** 10.1186/s13223-018-0317-y

**Published:** 2019-01-05

**Authors:** Che Kang Lim, Hassan Abolhassani, Sofia K. Appelberg, Mikael Sundin, Lennart Hammarström

**Affiliations:** 1 Division of Clinical Immunology and Transfusion Medicine, Department of Laboratory Medicine, Karolinska Institutet at Karolinska University Hospital Huddinge, 141 86, Stockholm, Sweden; 2 Department of Clinical Translational Research, Singapore General Hospital, Singapore, Singapore; 3 Research Center for Immunodeficiencies, Pediatrics Center of Excellence, Children’s Medical Center, Tehran University of Medical Sciences, Tehran, Iran; 4 Department of Blood Disorders, Immunodeficiency and Stem Cell Transplantation, Astrid Lindgren Children’s Hospital, Stockholm, Sweden; 5 Division of Pediatrics, Department of Clinical Science, Intervention and Technology, Karolinska Institutet, Stockholm, Sweden; 6 BGI-Shenzhen, Shenzhen, 518083 China

**Keywords:** Interleukin 2 receptor gamma, Atypical severe combined immunodeficiency, Hypomorphic mutations

## Abstract

**Background:**

Atypical X-linked severe combined immunodeficiency (X-SCID) is a variant of cellular immunodeficiency due to hypomorphic mutations in the interleukin 2 receptor gamma (*IL2RG*) gene. Due to a leaky clinical phenotype, diagnosis and appropriate treatment are challenging in these patients.

**Case presentation:**

We report a 16-year-old patient with a T^low^ B^+^ NK^+^ cellular immunodeficiency due to a novel nonsense mutation in exon 8 (p.R328X) of the *IL2RG* gene. Functional impairment of the *IL2RG* was confirmed by IL2-Janus kinase 3-signal transducer and activator of transcription signaling pathway investigation. In addition, the characteristics of the mutations previously described in 39 patients with an atypical phenotype were reviewed and analyzed from the literature.

**Conclusion:**

This is the first report of an atypical X-SCID phenotype due to an exon 8 mutation in the *IL2RG* gene. The variability in the phenotypic spectrum of classic X-SCID associated gene highlights the necessity of multi-disciplinary cooperation vigilance for a more accurate diagnostic workup.

**Electronic supplementary material:**

The online version of this article (10.1186/s13223-018-0317-y) contains supplementary material, which is available to authorized users.

## Background

Interleukin 2 receptor gamma (IL2RG) is an important signaling component for IL2, IL4, IL7, IL9, IL15, and IL21 [1]. The gene encodes a common gamma chain (γC) that is essential in the ontogeny and function of immune cells, in particular T and NK cells. Mutations in the gene result in X-linked severe combined immunodeficiency (X-SCID) [2].

Approximately 200 unique mutations in the *IL2RG* gene have been identified to date in more than 320 patients with X-SCID. Missense and nonsense mutations comprise around 48%, while insertion/deletion and splicing mutations account for 29% and 23%, respectively according to the mutation database (Table 1). The mutations lead to the production of a nonfunctional γC or prevent the protein from being produced, resulting in an arrest in lymphocyte development.

**Table 1:** Summary of *IL2 RG* unique mutation* and comparison with hypomorphic mutations

Parameters	Total unique mutation	Total observed	Typical SCID	Atypical SCID	*p* value
Exons					
Exon 1	12	15	13	2	0.6642
Exon 2	23	28	28	0	0.0574
Exon 3	47	72	68	4	0.1377
Exon 4	38	47	45	2	0.1998
Exon 5	38	106	87	19	0.0046**
Exon 6	19	36	34	2	0.4018
Exon 7	14	41	32	9	0.0253**
Exon 8	9	11	10	1^a^	1.0000
Others^b^	3	6	6	0	1.0000
Type of mutation					
Missense	61	138	108	30	< 0.0001**
Nonsense	36	73	70	3	0.0533
Insertion	15	21	20	1	0.7113
Deletion	43	52	50	2	0.1386
Splicing	35	62	59	3	0.1692
Others^c^	13	16	16	0	0.3929
Total	202	362	323	39	

*Summary based on NCBI Clinvar database (http://www.ncbi.nlm.nih.gov/clinvar/), LOVD gene database [25] (http://www.ncbi.nlm.nih.gov/lovd/home.php?select_db=IL2RG) and OMIM database (http://www.omim.org/)

**Fisher’s exact test was used to analyze the association of exons or mutation type in the distribution of different clinical phenotypes (Typical SCID vs Atypical SCID) observed and p ≤ 0.05 was regarded as significant

^a^Patient in the present study

^b^Includes large deletions

^c^Includes complex mutations, disruption of poly-A addition, variants within the first codon

In typical X-SCID, the disease is characterized by an almost complete absence of T and NK cells, and nearly normal or high numbers of functionally deficient B cells (T^–^B^+^NK^−^ phenotype). Infants with X-SCID are highly susceptibility to bacterial and opportunistic infections. Additional features include protracted diarrhea, rash, fever, pneumonia and sepsis. The disease is usually lethal within the 1st year of life unless reconstitution of the immune system is carried out.

Different mutations in the *IL2RG* gene have also been shown to be associated with less severe phenotypic variants. Several patients having hypomorphic *IL2RG* mutation with a milder form of combined immunodeficiency, termed “atypical X-SCID”, have been described previously [3–9]. Some patients might be less susceptible to infections, the reduction of T cells is relatively moderate and a normal lymphocyte proliferation assay may be observed. In addition, some of these patients might not be detected by newborn screening programs for SCID [10].

Due to poorly defined clinical and immunological phenotypes, the diagnosis is usually established later in childhood or even in adulthood, and the appropriate treatment is thus delayed.

Here we describe a novel nonsense mutation in the *IL2RG* gene consisting of a single nucleotide substitution at exon 8, in which a normal count of NK cells was found in peripheral blood.

## Case presentation

**Fig. 1:**
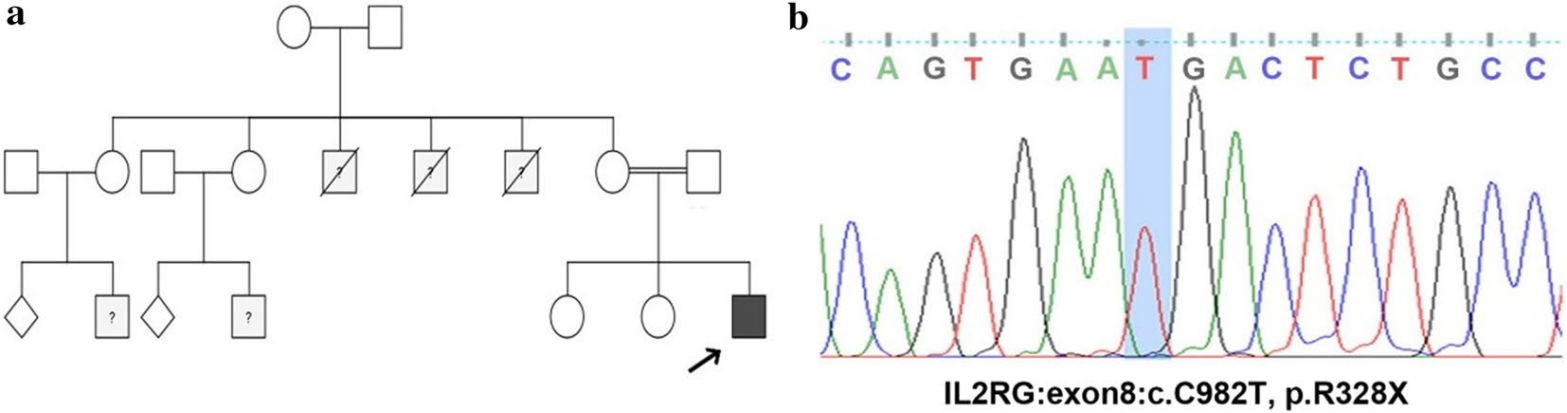
**a** Family pedigree of the patient(s). **b** Sanger sequencing

The patient, a 16 years old male of Kurdish ethnicity, was admitted to the pediatric lung and allergy service of Astrid Lindgren Children’s Hospital at Karolinska University Hospital due to chronic airway hypersensitivity and recurrent sinopulmonary infections. He is the third child of consanguineous parents with a family history of several early deaths due to lung failure on the maternal side (Fig. 1). He had a normal vaccination history but a medical history of four hospitalizations due to enteroviral infection (at age 16 months presenting with skin rash and diarrhea), chronic cough and fever (at age 18 months due to *Moraxella catarrhalis*), otitis media, adenopathy and shingles (leading to tympanostomy at the age of 2), pneumonia and an asthmatic reaction (at the age of 6).

At the age of 8 years, a computed tomography was performed due to a progression of his pulmonary disease which revealed bronchiectasis and a right middle lobe atelectasis. Immunologic profiles were investigated as previously described [11]. Although a complete blood count and immunoglobulin levels were normal, lymphocyte populations were measured. Low CD4^+^ and CD8^+^ T cell numbers, with normal numbers of B and NK cells were detected (Fig. 1 and Table 2). The patient had low specific cell-mediated immune response in activated whole blood using mitogens and antigen, such as pokeweed mitogen (PWM), candida antigen etc. (Table 3). However, normal response to mitogens phytohemagglutinin (PHA) and concanavalin A (Con A) by CD4^+^ T cells, but not CD8^+^ T cells were detected. The observation suggested that PHA and ConA stimulations for CD4^+^ T cells may be different from CD8^+^ T cells. Despite his combined immunodeficiency, the patient was free from opportunistic infections and his condition improved with temporary substitution of subcutaneous immunoglobulin and prophylactic antibiotics.

**Table 2:** Immunologic characteristics of the patient with hypomorphic/atypical X-linked severe combined immunodeficiency

Parameters	2004^a^ (5 years)	2010^a, b^ (8 years)	2011^c^ (9 years)	2012 (10 years)	Normal range
White blood cells (× 10^3^ cells/µL)	17↑	15↑	6.5	7.2	5.0–13.0
Absolute number of lymphocytes (× 10^3^ cells/µL)	4.8	1.9	1.3↓	1.3↓	1.5–6.5
Absolute number of neutrophils (× 10^3^ cells/µL)	11↑	12↑	4.4	4.7	2.0–8.4
Absolute number of monocytes (cells/µL)	760	900↑	700	700	200–800
Absolute CD3^+^ (cells/µL)	–	100↓	270↓	310↓	800–3500
Absolute CD3^+^CD4^+^ (cells/µL)	–	60↓	140↓	150↓	300–2000
Absolute CD3^+^CD8^+^ (cells/µL)	–	40↓	120↓	130↓	300–1800
CD4/CD8 ratio	–	1.5	1.1	1.1	0.9–2.6
Absolute CD19^+^ (cells/µL)	–	100↓	240	360	200–600
Absolute CD16/56^+^ (cells/µL)	–	890	690	1040	70–1200
IgG (mg/dL)	–	1150	1050	1130	610–1450
IgG1 (mg/dL)	–	–	711	–	350–910
IgG2 (mg/dL)	–	–	165	–	85–330
IgG3 (mg/dL)	–	–	146	–	20–104
IgA (mg/dL)	–	127	NI	77	70–365
IgM (mg/dL)	–	115	NI	85	24–210

*NI* Not investigated

^a^Measurement at the time of an acute infection

^b^IgG were measured before receiving subcutaneous IgG replacement therapy

^c^IgG subclasses values were measured after 1 month withdrawal of subcutaneous IgG replacement therapy at the age of 9

**Table 3:** Proliferation and specific antibody tests of the patient with hypomorphic/atypical X-linked severe combined immunodeficiency

Parameters	Result	Year (age)	Normal range
CD4 PHA (c/µL)	293	2012 (10 years)	170–3499
CD8 PHA (c/µL)	16↓	2012 (10 years)	76–3640
CD4 ConA (c/µL)	616↓	2012 (10 years)	620–3800
CD8 ConA (c/µL)	44↓	2012 (10 years)	180–1757
CD4 PWM (c/µL)	241	2012 (10 years)	233–2189
CD8 PWM (c/µL)	15↓	2012 (10 years)	50–549
CD19 PWM (c/µL)	61	2012 (10 years)	42–741
CD4 Staph enterotoxin (c/µL)	1242	2012 (10 years)	553–7746
CD8 Staph enterotoxin (c/µL)	275	2012 (10 years)	123–2365
CD4 Influenza (c/µL)	136	2012 (10 years)	19–1050
CD8 Influenza (c/µL)	0↓	2012 (10 years)	5–2020
CD4 Tetanus toxin (c/µL)	0↓	2012 (10 years)	5–306
CD8 Tetanus toxin (c/µL)	0↓	2012 (10 years)	5–14
CD4 PPD (c/µL)	0↓	2012 (10 years)	11–14
CD8 PPD (c/µL)	0↓	2012 (10 years)	5–29
CD4 Candida (c/µL)	5↓	2012 (10 years)	51–1014
CD8 Candida (CD4, CD8)	0↓	2012 (10 years)	5–49
CD4 Pneumococcus (c/µL)	2↓	2012 (10 years)	5–269
CD8 Pneumococcus (c/µL)	0↓	2012 (10 years)	5–13
CD4 Varicella zoster (c/µL)	45	2012 (10 years)	5–157
CD8 Varicella zoster (c/µL)	4↓	2012 (10 years)	5–23
Anti diphtheria (IgG, IU/mL)	0.53	2010 (8 years)^a^	0.1–56.2
Anti tetanus (IgG, IU/mL)	1.4	2010 (8 years)^a^	0.09–12.87
Anti tetanus (IgG1, mg/L)	17	2010 (8 years)^a^	0.9–228.5
Anti *Haemophilus influenzae* B (IgG, mg/L)	1.5	2010 (8 years)^a^	0.15–29.5
Anti PPV–23 (IgG, mg/L)	45	2010 (8 years)^a^	9.2–22.5
Anti PPV–23 (IgG2, mg/L)	25	2010 (8 years)^a^	0.8–122.4
Anti CMV (IgG, EIA, titer)	21,000↑	2011 (9 years)^a^	0–300
Anti CMV (IgM, EIA, titer)	0	2011 (9 years)^a^	0–10
Anti EBV EBNA (IgG, EIA, U/mL)	10	2011 (9 years)^a^	0–20
Anti EBV VCA (IgM, EIA, titer)	17	2011 (9 years)^a^	0–25
Anti HSV (IgG, EIA, titer)	150	2011 (9 years)^a^	0–230
Anti VZV (IgG, EIA, titer)	2600↑	2011 (9 years)^a^	0–350
Polio virus 1,2,3 neutralization test	512,128, 2048	2011 (9 years)^a^	300–3800
Anti measles (IgG, EIA, titer)	1500	2011 (9 years)^a^	500–2500
Anti Helicobacter pylori (IgG, EIA, titer)	0	2010 (8 years)^a^	0–50

Assays were performed in the Karolinska University Hospital according to the method described previously [26]

*PHA* phytohemagglutinin test, *ConA* concanavalin A, *PWM* pokeweed mitogen, *PPV-23*: Pneumococcal polysaccharide vaccine Pneumovax23, *CMV*: Cytomegalovirus, *EBV*: Epstein–Barr virus, *EBNA*: EBV nuclear antigen, *VCA*: viral capsid antigen, *HSV*: Herpes simplex virus, *VZV*: varicella-zoster virus, *PPD*: Purified protein derivative

^a^All specific antibodies tested at the age of 8 was before IgG replacement and at the age of 9 after 1 month withdrawal of IgG replacement

In order to identify the molecular defect, whole exome sequencing (WES) was performed. As the patient was born in a consanguineous family and showed a family history of recurrent infections and early death on the maternal side, an autosomal recessive or X-linked inheritances pattern was expected. Analysis of all variants were performed according to a standard pipeline described previously [12]; we identified 2 homozygous (autosomal) and 5 hemizygous (X-linked) variants which were absent from dbSNP database and 1000 Genome database (Additional file 1: Table S1). Comparing with the primary immunodeficiency genes database, the only variant consistent with the patient’s immunological phenotype was a novel nonsense mutation, p.R328X (c.982C>T) in exon 8 of the *IL2RG* gene (Fig. 1). Based on this finding, the therapeutic plan of the patient was changed and he became a potential candidate for allogeneic hematopoietic stem cell transplantation.

**Fig. 2:**
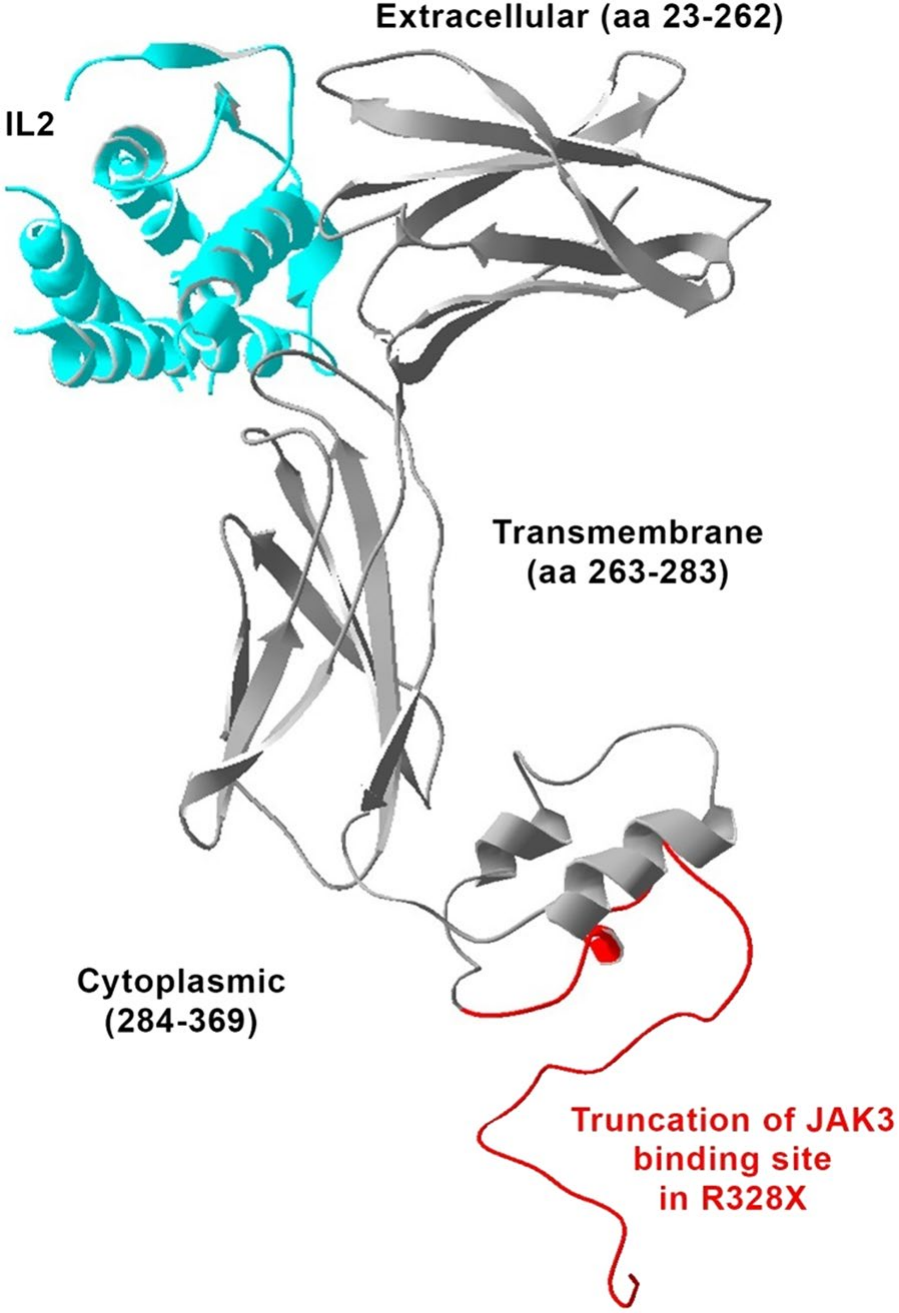
Schematic representation of the IL-2 receptor with a 42 amino-acids deletion of the intracellular domain of the γC shown in red

**Fig. 3:**
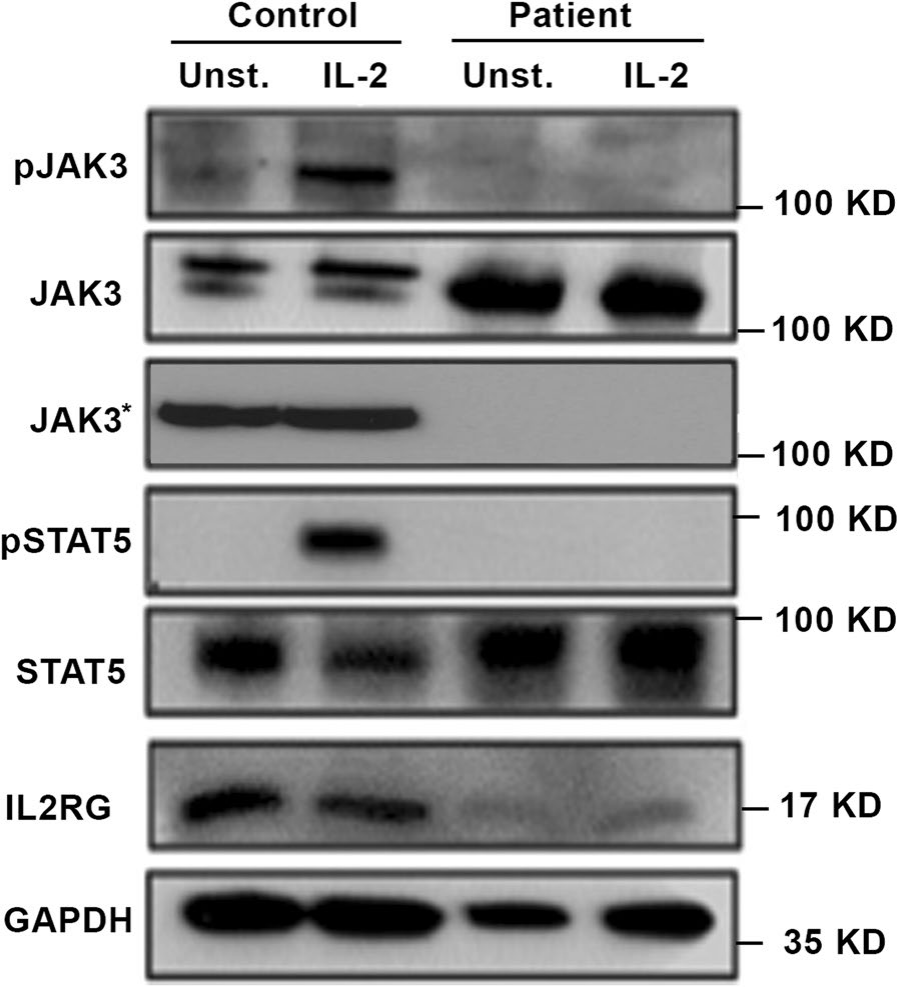
JAK3-STAT5 signaling pathway in patients with atypical X-SCID. Expression of activated JAK3 and STAT5, as well as total JAK3, JAK3* (reprobe with anti-JAK3 antibody binding to the kinase domain), STAT5, IL2R and GAPDH in unstimulated or IL2 stimulated cells, were evaluated by Western blot

Since the mutation causes a 42 amino acid truncation of the intracellular domain of the γC, including of the Janus kinase 3 (JAK3) binding site (Fig. 2), we investigated the expression of members of the IL2/JAK3 signaling pathway by western blot. Western blot (Fig. 3) demonstrated absence of IL2RG, suggesting that the mutation caused degradation of the molecule. In addition, IL2 stimulation activated JAK3 and signal transducer and activator of transcription signaling 5 (STAT5) proteins in cells from a healthy control but no activation was observed in the patient; indicating an impairment of IL-2 signaling. STAT5 expression was observed in both the control and the patient, while the main JAK3 isoform (1124 amino acids, 115kDA) was only observed in the control. However, the intensity of second isoform of JAK3 (1094 amino acids, lacking part of the kinase domain) was stronger in the patient. When the blot was reprobed with another anti-JAK3 antibody (binding to the kinase domain), expression were observed only in the control samples, indicating that the expression pattern of JAK3 is modified in the patient (Fig. 3).

## Discussion and conclusions

Approximately 10% of the reported *IL2RG* mutations have been associated with atypical phenotypic variants (including T^low/−^ B^+^ NK^+^ and T^low/−^ B^low^ NK^+/low/−^, Table 1). The majority of the atypical patients present a “milder” form of immunodeficiency. Database summary shows that nearly half of the total *IL2RG* mutations are located in exon 5 (29.4%) and exon 3 (19.9%). Mutations in exon 5, which encodes the extracellular domain including the highly conserved WSXWS motif: a region essential for proper protein folding and thereby efficient intracellular transport as well as extracellular receptor binding, are expected to disrupt the γC configuration. Likewise, a similar clinically severe effect is predicted when the mutations occur in exon 3, which will modify the amino acids directly or close to the four conserved cysteine residues.

**Fig. 4:**
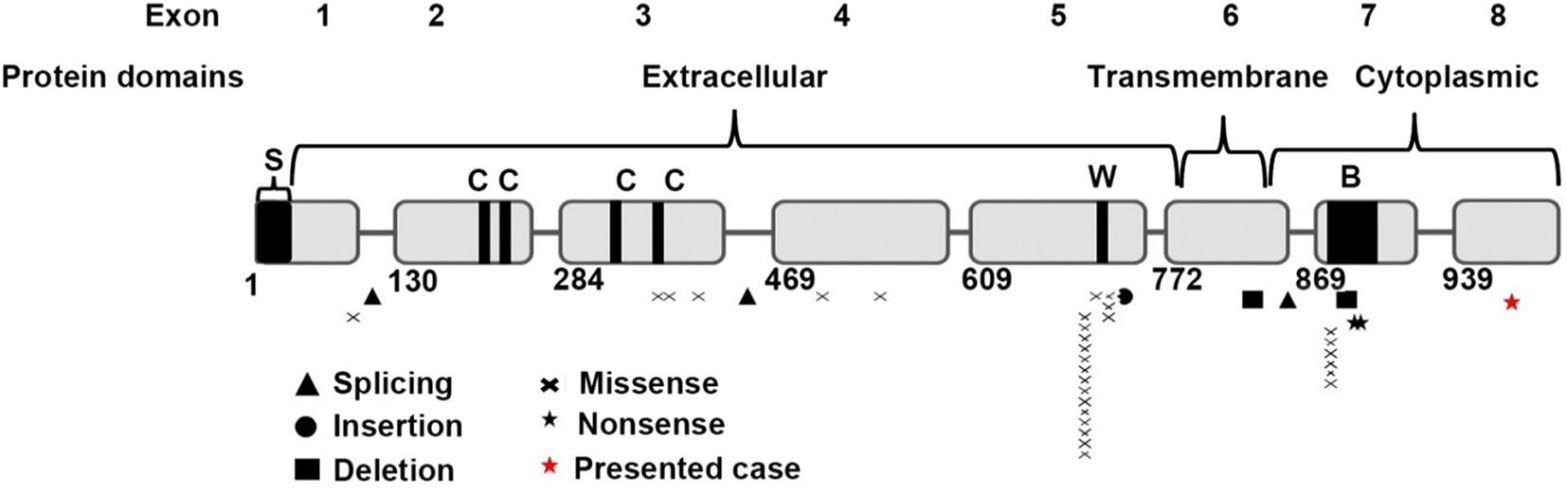
*IL2RG* gene location of pathogenic hypomorphic mutations [27, 28] in 39 reported atypical X-SCID patients [1, 4, 6, 7, 9, 15, 17, 21, 22, 29–36] (S: Signaling sequence; C: Conserved cysteine residues, W: WSXWS box; B: box1/box2 domain)

Mutation in exons 5 and 7, on the other hand, are significantly associated with atypical X-SCID (Fig. 4). Mutation of p.R222C in exon 5 (14 cases) is the most frequent variants among patients with NK^+^ phenotype [6] which leads to a differential impairment of cytokines pathways (IL21 > IL15/IL2 > IL4). Furthermore, this mutation leads to a defect in lymphocyte function rather than immune cell development since normal differentiation of lymphocytes (B and T cells) as well as a normal thymus gland has been observed [8].

Mutations in exon 8 are relatively rare (~ 3% including the present case). Our patient is the first atypical X-SCID with an exon 8 mutation while nine cases were reported with a mutation in exon 7 (Table 1). Exon 7 and exon 8 encode the intracellular domains of *IL2RG*. The exon 8 nonsense mutation in our patient results in a truncation of 42 amino acids in the cytoplasmic domain of the γC. The mutation putatively reduced the binding site of JAK3 (Fig. 2), most likely preventing the association of JAK3 with the γC. In line with this finding, Hofman et al. 2004 showed that JAK3 is dependent on γC binding for membrane localization and in the absence of IL2R, JAK3 ends up in the cytosol [13]. Moreover, Jayshree et al. [14] demonstrated that IL2 regulates the transcription of *JAK3* through the concentration-dependent effects of phosphorylation-dephosphorylation of JAK3. The truncation of γC abolished the JAK3 binding site and resulted in no or low phosphorylation of JAK3. Accumulation of low or unphosphorylated JAK3 may thus modify its own transcription and hence downregulate the expression of main hematopoietic isoform JAK3. Previous reports have also described patients with atypical X-SCID with a mutation in the cytoplasmic domain of γC [3, 15]. The mutation in exon 7 (p.L293Q) impaired the association with *JAK3* [16]. On the contrary, multiple cases of typical SCID phenotype have been observed with mutations in exon 8 which result in the truncation of the last 45 [17], 48 amino acids [18, 19] as well as substitution of 40 amino acids [18] and 56 amino acids [20] in the cytoplasmic domain. The discrepancies of the clinical features in patients with cytoplasmic domain mutations suggest that the level of interaction between γC and JAK3 may play an important role in defining the phenotypic manifestations.

NK+ phenotype was mainly observed in patients having mutation in exon 3 [21, 22], exon 5 [6, 18] and exon 7 [15]. Our patient is the first NK+ phenotype with an exon 8 mutation. The residual population of NK cells in selected patients with γC deficient may be due to the contribution of IL12 to IL15-independent NK cell expansion. This critical role of IL12 is evident not only by its effect on IFN-γ production by NK cells and NK cell blastogenesis during viral infections, but also in the *Ilr2g*^−*/*−^ mouse model, where a 30-fold expansion of the absolute number of Ly49H^+^KLRG1^+^ NK cells has been noted [23]. On the other hand, increasing concentrations of dysfunctional γC due to a hypomorphic mutation could partly compensate STAT5 phosphorylation after IL15 stimulation, but not after IL7 stimulation [24]. Therefore, the presence of an NK^+^ phenotype in the patient suggests that the underlying *IL2RG* mutation results in a preferential retention of IL15 mediated signaling.

Delayed diagnostic as well as limited sample material has prevented us from investigating whether there is prolonged transplacental maternal T cell engraftment. Further investigation is required to elucidate the relationship between the mutations of the γC and clinical manifestations in patients with X-SCID to achieve a better classification of the disease. In addition, the heterogeneity of clinical presentation in primary immunodeficient patients highlights the role of a more accurate diagnostic work up with the aid of multi-omics molecular diagnostics.

## Additional file

**Additional file 1: Table S1.** Homozygous (autosomal) and hemizygous (X-linked) variants identified by whole exome sequencing. **Table S2.** Summary of genetic and clinical characteristics of patients with hypomorphic/atypical X-linked severe combined immunodeficiency.

## Abbreviations

X-SCID: X-linked atypical severe combined immunodeficiency
IL2RG: interleukin 2 receptor gamma
γC: common gamma chain
PHA: phytohemagglutinin
Con A: concanavalin A
PWM: pokeweed mitogen
WES: whole exome sequencing
JAK3: Janus kinase 3
STAT5: signal transducer and activator of transcription signaling 5
