# Supplementary material for: IL2RG hypomorphic mutation: identification of a novel pathogenic mutation in exon 8 and a review of the literature
Source: Allergy Asthma Clin Immunol. 2019 Jan 5;15:2. doi: 10.1186/s13223-018-0317-y (PMC6320602; doi:10.1186/s13223-018-0317-y)
Supplement: Supplementary file 1 — Additional file 1: Table S1. Homozygous (autosomal) and hemizygous (X-linked) variants identified by whole exome sequencing. Table S2. Summary of genetic and clinical characteristics of patients with hypomorphic/atypical X-linked severe combined immunodeficiency. [file 13223_2018_317_MOESM1_ESM.docx]

**Additional data**

***IL2RG* Hypomorphic Mutation: Identification of a Novel Pathogenic Mutation in Exon8 and a Review of the Literature**

**Lim et al.**

**Table S1.** Homozygous (autosomal) and hemizygous (X-linked) variants identified by whole exome sequencing

| **Chromosome** | **Genomic Position** | **Reference Allele** | **Alternative Allele** | **Gene** | **cDNA change** | **Protein change** | **Allele frequency** | | | **CADD score** | **MSC-CADD Score^b^** | **MSC-CADD Impact Prediction^b^** |
| --- | --- | --- | --- | --- | --- | --- | --- | --- | --- | --- | --- | --- |
|  |  |  |  |  |  |  | **ESP6500** | **1000 Genome** | **Exac^a^ nontcga** |  |  |  |
| chr3 | 195512042 | T | C | *MUC4* | c.A6409G | p.T2137A | NA | NA | 9.09E-01 | 0.04 | 14.40 | low |
| chr21 | 14982904 | G | A | *POTED* | c.G355A | p.A119T | NA | NA | 3.04E-05 | 0.02 | 14.40 | low |
| chrX | 67941383 | C | T | *STARD8* | c.C2014T | p.P672S | NA | NA | 1.37E-05 | 24.20 | 14.40 | high |
| chrX | 69478531 | C | T | *P2RY4* | c.G944A | p.R315H | NA | NA | 1.37E-05 | 23.30 | 14.40 | high |
| chrX | 70327714 | G | A | *IL2RG* | c.C982T | p.R328X | NA | NA | NA | 37.00 | 22.80 | high |
| chrX | 131207022 | A | G | *MST4* | c.A941G | p.Q314R | NA | NA | 4.00E-05 | 16.60 | 24.58 | low |
| chrX | 153670018 | C | T | *GDI1* | c.C868T | p.R290C | NA | NA | NA | 27.60 | 12.76 | high |

*^a^Allele frequency from Exome Aggregation Consortium (*ExAC*)*non-TCGA (germline only)

*^b^The Mutation Significance Cutoff (MSC) for the Combined Annotation Dependent Depletion (CADD)[27] score for each variant was estimated by MSC server[28] at confidence interval of 99%.*

**Table S2.** Summary of genetic and clinical characteristics of patients with hypomorphic/atypical X-linked severe combined immunodeficiency

| **No** | **Exon** | **Protein change** | **cDNA change** | **Type of Mutation** | **Phenotype** | **Age of onset** | **Clinical manifestation** | **Reference** |
| --- | --- | --- | --- | --- | --- | --- | --- | --- |
|  |  |  |  |  |  |  |  |  |
| 1 | 1 | D39N | c.129G>A | missense and disruption of RNA splicing | T^+^B^+^NK^+^ | 9 months | protracted diarrhea, failure to thrive | [[4](#_ENREF_4)] |
| 2 | 1 | splice | c.129+5G>A | disruption of RNA splicing | T^+^B^+^NK^+^ | 5 months | [protracted diarrhea, failure to thrive, hypoproteinemia, erythematous rash, alopecia, persistent cough, fever, hepatosplenomegaly, and lymphadenopathy.](http://www.bloodjournal.org/content/112/5/1872#F1) | [[29](#_ENREF_29)] |
| 3 | 3 | G114A | c.341G>C | missense | T^+^B^+^NK^+^ | 7 months | oral thrush, otitis media, pneumonia and genitourinary diaper dermatitis | [[21](#_ENREF_21)] |
| 4 | 3 | C115R | c.343T>C | missense | T^+^B^high^NK^-^ | 6 months | severe interstitial pneumonia, hypogammaglobulinemia | [[30](#_ENREF_30)] |
| 5 | 3 | splice | c.468+3A>C | disruption of RNA splicing | T^-^B^+^NK^+^ | 6 months | recurrent infection and failure to thrive | [[22](#_ENREF_22)] |
| 6 | 3 | L146P | c.451T>C | missense | T^low^B^+^NK^low^ | 14 months | recurrent infection since 14 months | [[17](#_ENREF_17)] |
| 7 | 4 | L162R | c.499T>G | missense | T^low^B^+^NK^low^ | 3 months | interstitial pneumonia, failure to thrive, persistent oral candidiasis | [[7](#_ENREF_7)] |
| 8 | 4 | L183S | c.562T>C | missense | T^+^B^+^NK^-^ | 4 months | pneumocystis carinii infection and persistence infectious problem | [[9](#_ENREF_9)] |
| 9 | 5 | R222C | c.678C>T | missense | T^+^B^+^NK^+^ | - | disseminated cytomegalovirus (CMV) infection, severe neurological deterioration, oral and perineal candidiasis, chronic diarrhea, and failure to grow, | [[7](#_ENREF_7)] |
| 10 | 5 | R222C | c.678C>T | missense | T^+^B^+^NK^+^ | - | pneumocystic carinii infection. Grew normally | [[8](#_ENREF_8)] |
| 11 | 5 | R222C | c.678C>T | missense | T^+^B^+^NK^+^ | 10 months | disseminated cytomegalovirus infection | [[6](#_ENREF_6)] |
| 12 | 5 | R222C | c.678C>T | missense | T^low^B^+^NK^+^ | 7 months | pneumocystis jirovecii infection | [[6](#_ENREF_6)] |
| 13 | 5 | R222C | c.678C>T | missense | T^low^B^+^NK^+^ | 6 months | pneumocystis jirovecii infection | [[6](#_ENREF_6)] |
| 14 | 5 | R222C | c.678C>T | missense | T^low^B^+^NK^+^ | 7 months | disseminated adenovirus infection | [[6](#_ENREF_6)] |
| 15 | 5 | R222C | c.678C>T | missense | T^low^B^+^NK^+^ | 4 months | pneumocystis jirovecii, respiratory syncytial virus infection | [[6](#_ENREF_6)] |
| 16 | 5 | R222C | c.678C>T | missense | T^low^B^+^NK^+^ | 6 months | pneumocystis jirovecii, disseminated cytomegalovirus infection | [[6](#_ENREF_6)] |
| 17 | 5 | R222C | c.678C>T | missense | T^low^B^+^NK^+^ | 7 months | pneumocystis jirovecii, disseminated cytomegalovirus infection | [[6](#_ENREF_6)] |
| 18 | 5 | R222C | c.678C>T | missense | T^low^B^+^NK^+^ | - | -^a^ | [[6](#_ENREF_6)] |
| 19 | 5 | R222C | c.678C>T | missense | T^+^B^+^NK^+^ | 2 months | pneumocystis jirovecii, failure to thrive, candida, skin rash | [[6](#_ENREF_6)] |
| 20 | 5 | R222C | c.678C>T | missense | T^low^B^+^NK^+^ | - | -^a^ | [[6](#_ENREF_6)] |
| 21 | 5 | R222C | c.678C>T | missense | T^+^B^+^NK^+^ | 6 months | interstitial pneumocystis carinii pneumonia, oral thrush and recurrent skin infections | [[31](#_ENREF_31)] |
| 22 | 5 | R222C | c.678C>T | missense | T^+^B^+^NK^+^ | 9 months | interstitial pneumocystis carinii pneumonia | [[31](#_ENREF_31)] |
| 23 | 5 | R226C | c.690C>T | missense | T^+^B^+^NK^low^ | - | - | [[1](#_ENREF_1)] |
| 24 | 5 | R226C | c.690C>T | missense | T^-^B^-^NK^+^ | - | - | [[18](#_ENREF_18)] |
| 25 | 5 | R226C | c.690C>T | missense | T^-^B^-^NK^-^ | - | - | [[18](#_ENREF_18)] |
| 26 | 5 | R226H | c.691G>A | missense | T^low^B^+^NK^+^ | - | - | [[1](#_ENREF_1)] |
| 27 | 5 | H242_P243insH | c.740insACC | insertion | T^-^B^+^NK^+^ | 5 months | protracted diarrhea and failure to thrive. Staphylococcus auricularis, Staphylococcus epidermidis, and Candida parapsilopsis (blood) infections. Candida albicans and Pseudomonas aeruginosa (stools) infections | [[32](#_ENREF_32)] |
| 28 | 6 | R285 | c.868G>A | disruption of RNA splicing | T^-^B^-^NK^+^ | 5 months | severe interstitial pneumonitis, poor feeding, coughing, vomiting and poor weight gain. | [[33](#_ENREF_33)] |
| 29 | 6 | L272fs | c.830del4 | deletion, frameshift | T^low^B^+^NK^low^ | 3 months | recurrent oral moniliasis and impetigo, chronic diarrhea and a respiratory tract infection, failure to thrive | [[34](#_ENREF_34)] |
| 30 | 7 | L293Q | c.892T>A | missense | T^+^B^+^NK^+^ | - | - | [[15](#_ENREF_15)] |
| 31 | 7 | L293Q | c.892T>A | missense | T^+^B^+^NK^+^ | - | - | [[15](#_ENREF_15)] |
| 32 | 7 | L293Q | c.892T>A | missense | T^+^B^+^NK^+^ | - | - | [[15](#_ENREF_15)] |
| 33 | 7 | L293Q | c.892T>A | missense | T^+^B^+^NK^+^ | - | - | [[15](#_ENREF_15)] |
| 34 | 7 | L293Q | c.892T>A | missense | T^+^B^+^NK^+^ | - | - | [[15](#_ENREF_15)] |
| 35 | 7 | L293Q | c.892T>A | missense | T^+^B^+^NK^+^ | - | - | [[15](#_ENREF_15)] |
| 36 | 7 | T301fs | c.916delC | deletion, frameshift | T^-^B^low^NK^-^ | - | - | [[35](#_ENREF_35)] |
| 37 | 7 | R289X | c.879C>T | nonsense | T^low^B^+^NK^low^ | - | - | [[1](#_ENREF_1)] |
| 38 | 7 | R289X | c.879C>T | nonsense | T^low^B^+^NK^low^ | 1 month | protracted diarrhea, failure to thrive, repeated bronchopneumonitis | [[36](#_ENREF_36)] |
| 39 | 8 | R328X | c.982C>T | nonsense | T^low^B^+^NK^+^ | 16 months | enteroviral infection,skin rash, diarrhea, chronic cough, fever, otitis media, adenopathy and shingles | ^b^ |

*^a^ Diagnosis at birth*

*^b^ Patient in the present study*
